# The role of IL-17-related signaling in myelomagenesis, disease prognosis/progression, and therapeutic approach—a scoping review

**DOI:** 10.1007/s10238-025-01728-6

**Published:** 2025-06-09

**Authors:** Piotr Kulig, Anna Rałowiec, Bartłomiej Baumert, Bogusław Machaliński

**Affiliations:** 1 Department of General Pathology, Pomeranian Medical University, Powstańców Wlkp. 72, 70-111 Szczecin, Poland; 2 Pharmaceutical Facility of Pomeranian Medical University, Plac Polskiego Czerwonego Krzyża 1, 71-899 Szczecin, Poland; 3 Department of Hematology and Transplantology, Pomeranian Medical University, Unii Lubelskiej 1, 71-252 Szczecin, Poland

**Keywords:** Multiple myeloma, IL-17, Immunotherapy, MM progression, Adjuvant therapy, Bone marrow microenvironment

## Abstract

Multiple myeloma (MM), a plasma cell malignancy, despite the progress in treatment, is still a challenge for both clinicians and affected patients. Modern treatment regimens including monoclonal antibodies, bispecific antibodies, CAR-T cell therapy as well as new generations of immunomodulatory drugs and proteasome inhibitors significantly improved clinical outcomes. Nonetheless, there is still a room for improvement and novel therapeutic strategies. IL-17-related signaling is a relatively undiscovered area in MM research. It was established that IL-17 is the growth factor for plasma cells including their malignant counterparts, is associated with myelomagenesis and disease progression. Furthermore, IL-17 axis can be pharmacologically targeted by monoclonal antibodies currently being used in different indications. In this narrative review we summarized the role of IL-17 axis in MM. Specifically, we focused on the role of IL-17 in MM development and progression with a particular emphasis upon clinical implications. Moreover, we have briefly summarized the potential role of therapeutic interference with IL-17-related singling and have outlined future research directions.

## Introduction

Multiple myeloma (MM) is a malignant tumor of plasma cells which, despite significant progress in its management, remains incurable. This disease is characterized by infiltration of the bone marrow (BM) by malignant plasma cells, which in the vast majority of cases secrete a monoclonal protein [1]. The symptomatology of MM is varied. Symptoms may be related to the disease itself or result from the treatment regimen used or its complications. The typical clinical picture of MM can be summarized by the acronym CRAB (C—calcium, hypercalcemia; R—renal, kidney failure; A—anemia; B—bone lesions) [2]. In addition, MM patients often report so-called “B” symptoms or constitutional symptoms (fever, night sweats, weight loss, and bone pain) caused by the release of proinflammatory cytokines and cancer-related hypermetabolism [3, 4]. In should be noted that bone pain may also be the result of osteolysis and pathological fractures [5].

Modern treatment strategies for MM involve combining drugs with different mechanisms of action. Majority of novel anti-MM agents harness the immune system to tackle the disease, resulting in longer survival and a better prognosis. However, despite recent advances in the treatment of MM, the disease remains incurable and the prognosis, although improved, is still not satisfactory. Disease relapse and refractoriness occur in vast majority of MM patients [6]. Identification of novel and potentially targetable molecular pathways involved in the promotion and progression of MM requires in-depth studies. Therefore, to better understand the disease and improve treatment outcomes of patients with MM, there is an urgent need to shed more light upon molecular pathways promoting myelomagenesis, the mechanisms of relapse and molecular background of refractoriness to the therapy.

One relatively unexplored area of ​​MM research is the role of IL-17-related signaling. IL-17 is a growth factor for plasma cells, including malignant MM cells [7, 8]. Moreover, it is associated with bone disease in MM [9]. Therefore, pharmacological interference with IL-17-related signaling seems reasonable and should be thoroughly investigated. Moreover, the results of our study revealed that MM patients who achieved a deep and long-lasting response to lenalidomide-based therapy had increased expression of the IL-17 receptor (IL-17R) compared to patients with newly diagnosed MM (NDMM) [10]. This finding suggests that MM cells, under the pressure exerted by LEN, seek to synthesize an IL-17-related signal, which is a growth factor for MM cells, presumably to avoid the anti-MM effects of the drug. On the other hand, this supports the hypothesis that therapeutic interference with IL-17-related signaling may serve as an effective adjuvant therapy, especially in the case of LEN-based treatment regimens, enhancing its cytotoxic effects on MM cells.

The aim of this narrative review is to shed further light on IL-17 signaling in the pathogenesis of MM. We also summarize the body of evidence that warrants further research, particularly aimed at investigating the potential utility of IL-17 antagonists in the treatment of MM.

## Search methodology

**Fig. 1:**
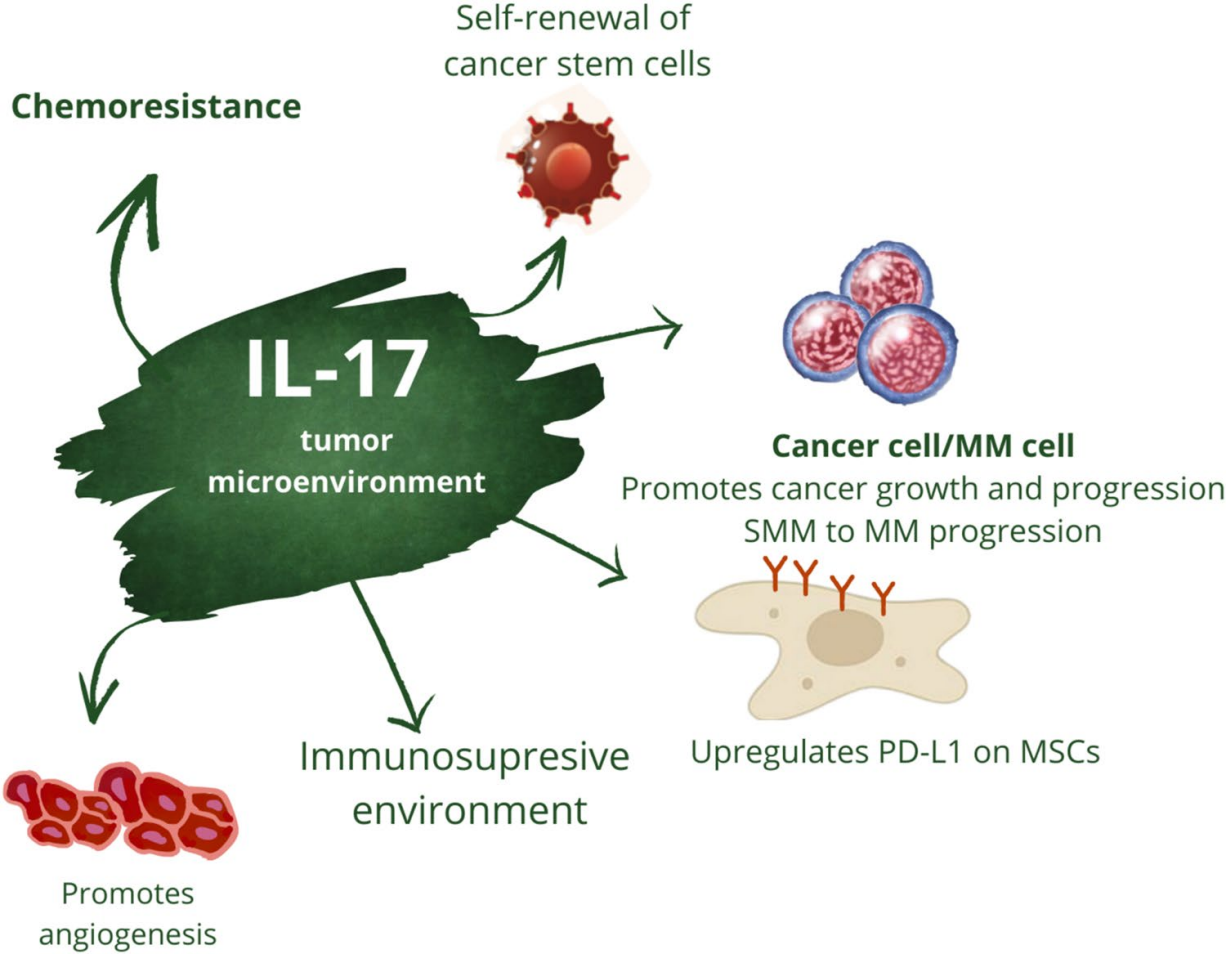
**IL-17 in tumor/MM microenvironment.** IL-17 shapes tumor niche in a multiple ways. Its complex interactions between stroma and malignant cells result in creation permissive environment for the malignancy necessary for disease promotion and progression. MSCs—mesenchymal stromal; cells/ PD-L1—programmed death-ligand 1; MM—multiple myeloma

**Fig. 2:**
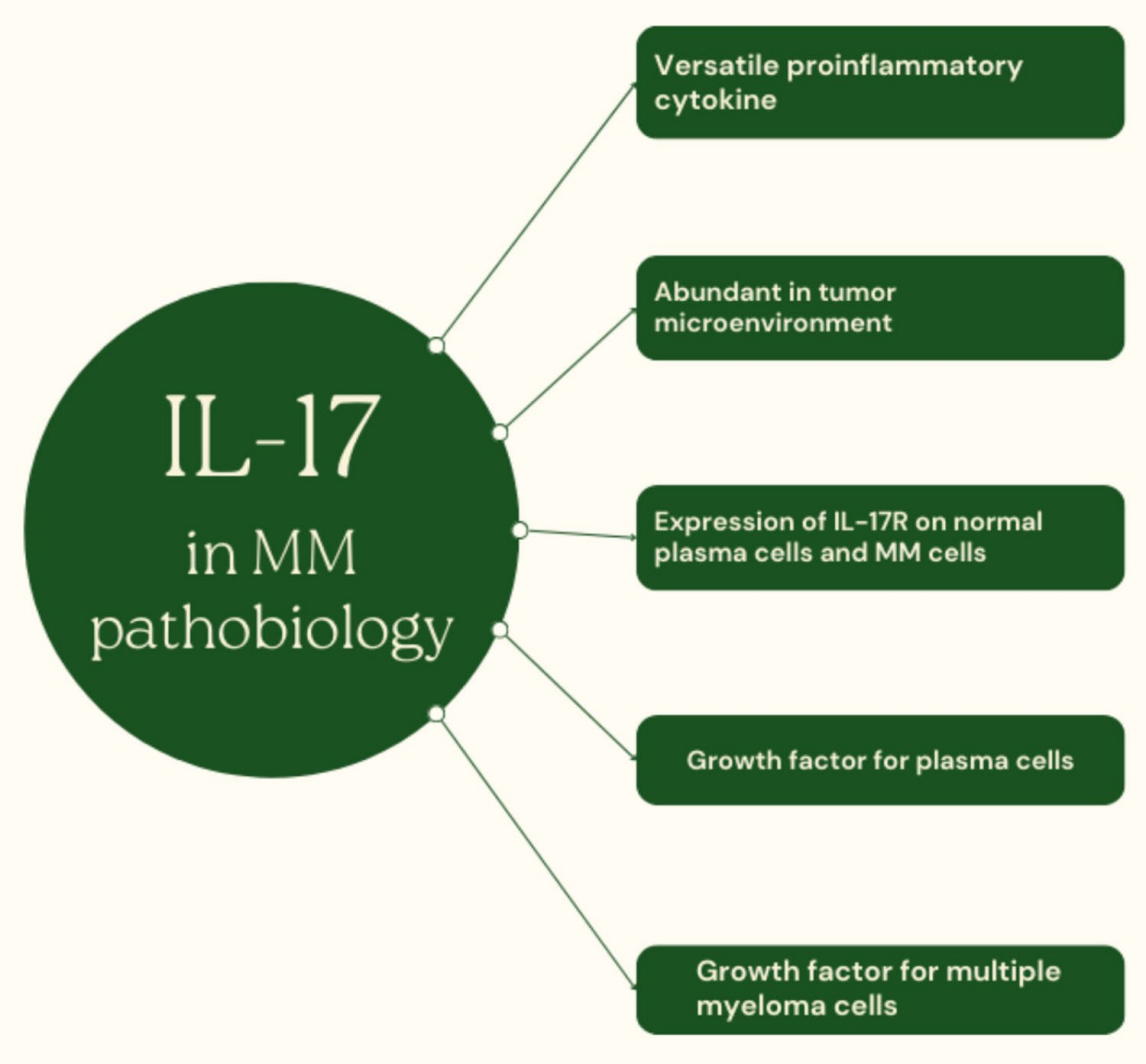
Role of the IL-17 axis in the pathobiology of multiple myeloma

**Fig. 3:**
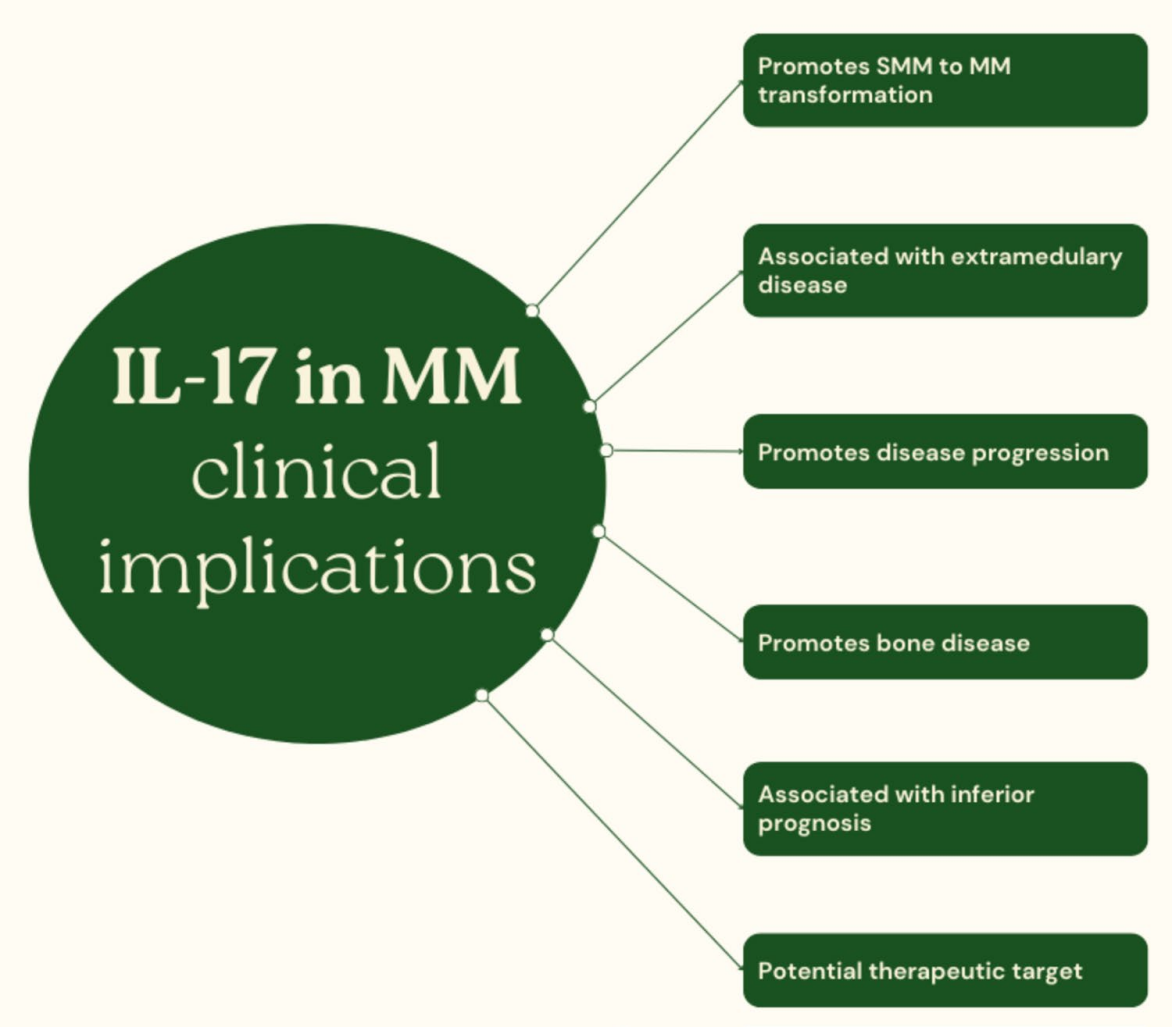
IL-17 in multiple myeloma—a brief overview of clinical implications

**Fig. 4:**
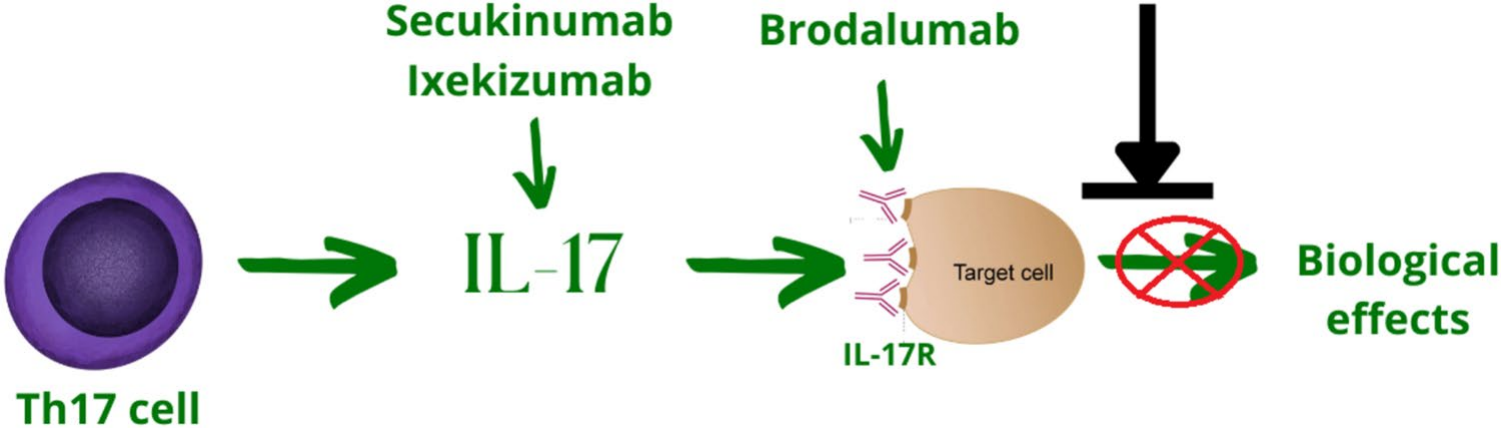
**Brief overview of IL-17 axis antagonists.** Secukinumab, Ixekizumab—monoclonal antibodies against IL-17A; Brodalumab—monoclonal antibody against IL-17 receptor. Secukinumab, Ixekizumab and Brodalumab are registered for the treatment of autoimmune disorders (psoriasis, psoriatic arthritis and ankylosing spondylitis) and are under preclinical investigation in MM

We searched the PubMed, PubMed Central, Scopus, Web of Science, Embase, and Google Scholar databases in detail for papers associated with MM, IL-17 and IL-17-related signaling. We used the following keywords: “multiple myeloma IL-17”; “multiple myeloma IL-17 axis”; “multiple myeloma Th17 cells”; “multiple myeloma IL-17 pathway” and “multiple myeloma IL-17 signaling”. In our review, we included all in vitro studies, clinical trials, and other types of studies, such as retrospective cohort studies, which were exclusively focused on multiple myeloma, myeloma cell lines, IL-17, IL-17 axis and IL-17-related signaling in Figs. 1, 2, 3 and 4.

## Multiple myeloma as a major challenge to healthcare systems

From an epidemiological point of view, MM poses a serious challenge to physicians and healthcare systems, as it is the second most common hematological malignancy and accounts for 1% of all cancers [11]. In the USA, the American Cancer Society estimates that in 2024, about 35,780 new cases will be diagnosed (19,520 in men and 16,260 in women) and the number of deaths will be about 12,540 (7,020 in men and 5,520 in women) [12]. The annual incidence is estimated at 7.1 per 100,000 men and women, and the mortality rate is 3.1 per 100,000 men and women. In 2020, the estimated total number of patients with MM in the USA was 170,405 [13]. The above epidemiological data imply that MM is a serious challenge for hematologists and healthcare systems worldwide. Given the recent advances in the treatment of MM, it can be assumed that the number of MM cases will continue to increase, thus increasing the burden on the healthcare system (Table 1).

**Table 1:** Summary of key studies

No	Author	Key findings	Ref
1	Yang et al	IL-17 promotes extramedullary disease in MM	[53]
2	Alexandriakis et al	IL-17 promotes angiogenesis in MM	[54]
3	Calcinotto et al	IL-17 promotes SMM to MM transition	[56]
4	Noonan et al	IL-17 promotes bone disease in MM	[9]
5	Wang et al	Percentage of Th17 cells within the BM is associated with disease stage	[62]
6	Song et al	Elevated levels of IL-17 negatively affect the prognosis of MM patients	[63]
7	Ma et al	Decreased Th 17 levels are associated with better response to MM treatment	[64]
8	Gu et al	IL-17A levels are correlated with poor OS and PFS in MM	[67]
9	Li et al	Elevated IL-17 levels negatively influenced treatment response and disease stage	[68]
10	Kulig et al	Deep response to Rd regimen is associated with IL-17R upregulation in MM cells	[10]

## Modern treatment of multiple myeloma as a double-edged sword: its potent antimyeloma effect prolongs survival but determines treatment limitations.

Untreated MM is an aggressive disease with poor survival and non-favorable clinical outcomes. However, with the introduction of new antimyeloma drugs, the prognosis for patients with multiple myeloma has improved significantly [14]. Virtually all novel anti-MM agents disrupt the immune system by hijacking its effector functions and redirecting them to counteract disease. Currently, triplet or quadruplet regimens (i.e., a combination of three or four drugs with different mechanisms of action) are standard treatment strategies as induction therapy [15, 16]. Several classes of drugs are available in modern MM therapy, and their introduction was a milestone in the treatment of this disease. The first breakthrough was the rediscovery of thalidomide (THAL), which became the first-in-class immunomodulating drug (IMiD) [17]. Its novel derivatives, lenalidomide (LEN) and pomalidomide (POM), are nowadays core compounds in MM treatment, widely incorporated in multiple treatment regimens [18]. The next significant milestone was the introduction of proteasome inhibitors (PIs), with bortezomib as the reference compound, which was then accompanied by newer molecules—carfilzomib and ixazomib [19]. Another new class of drugs are monoclonal antibodies, for example daratumumab, which is directed against CD38 [20]. More advanced immunotherapy includes bispecific antibodies and CAR-T cells, which, despite certain limitations, offer hope for further improvement of clinical outcomes [21, 22].

It should be emphasized that the implementation of novel anti-MM agents was the paramount factor in achieving longer survival. For example, according to the results of the cohort study by Kumar et al., the median overall survival (OS) for patients receiving at least one new drug as part of their initial therapy was not reached during the analyzed period (95% CI; 5.4, NR), compared with 3.8 years (95% CI; 3.2, 4.5) for those individuals not receiving a new drug as part of their initial therapy (*p* < 0.001). Multivariate analysis was then performed to determine the precise predictors of more favorable clinical outcomes. The results showed that only the use of new drugs was associated with improved survival, indicating that the improved OS in recent years can be mainly attributed to the increased use of new antimyeloma drugs as part of the induction regimen [23]. Currently, triplet or quadruplet regimens are standard treatment strategies as induction therapy [15, 16]. According to existing evidence, quadruple regimens are associated with a deeper response, with a comparable number of adverse events as triple regimens [24, 25]. It should be emphasized that prognosis and survival correlate positively with the quality of response. In particular, minimal residual disease (MRD) negativity is associated with favorable progression-free survival (PFS) and OS [26].

On the one hand, novel anti-MM agents have led to improved clinical outcomes, as exemplified by PFS and OS. On the other hand, their implementation is limited by adverse reactions, which in some individuals result in either dose reduction or even treatment discontinuation [27, 28]. Another reason for treatment cessation is disease progression with subsequent conversion to the next line of therapy [29]. It should be emphasized that disease relapse, especially early one, is a prognostic factor for worse clinical outcomes in subsequent lines of therapy [30]. Particularly clinical progression is associated with inferior survival, whereas MM patients with biochemical progression tend to have slightly better post-progression clinical outcomes [31]. The most serious adverse events in anti-MM therapy are peripheral neuropathy, hematological toxicity resulting in anemia, neutropenia or thrombocytopenia, infections and thromboembolic complications [32].

As mentioned above, adverse drug reactions, with particular emphasis on neuropathy and hematologic toxicity, often limit the use of new antimyeloma agents, which may negatively impact clinical outcomes. Therefore, there is an urgent need to develop and implement new treatment regimens or complementary therapies that can enhance the antimyeloma activity of currently used compounds without causing most clinically significant complications. Many new treatment strategies for MM are currently being investigated. The most important are bispecific antibodies [21], drug-antibody conjugates [33], CAR-T cells and CAR-NK cells [22]. Additionally, attempts are being made to interfere with the apoptosis pathways. For example, the BCL-2 inhibitor venetoclax has been shown to improve OS and PFS in individuals with MM, particularly with t(11;14) [34]. It should be noted, however, that the implementation of the above therapies, at least to some extent, is limited by side effects.

## Interleukin 17—an underestimated target for novel treatment strategies to tackle multiple myeloma?

### A brief overview of IL-17-related signaling

Harnessing the immune system to fight MM has proven to be an effective treatment method [35]. Therefore, it seems reasonable to search for potential new treatment strategies in the field of immune system modulation. There are molecular pathways that have not yet been thoroughly investigated in the context of MM and yet may become new targets for anti-MM treatment regimens. Particular attention should be paid to IL-17 due to its pleiotropic effects on, among others, plasma cells [7, 8]. Moreover, there are monoclonal antibodies directed against interleukin itself or its receptor.

This determines that IL-17-related signaling is potentially targetable in MM once the rationale for such treatment is provided first in preclinical models and subsequently in clinical trials. IL-17 is a universal proinflammatory cytokine secreted by Th17 cells [36]. Currently, it is well established that a magnitude of biological processes, including host defense against microbes, autoimmune reactions, metabolic reprogramming of lymphoid tissue, tissue repair and regeneration, and tumor promotion and progression, are attributed to IL-17-related signaling [37]. IL-17A was initially discovered and became the founding member of the IL-17 cytokine family. Subsequently, five additional molecules were identified, and the IL-17 cytokine family consists of a subset of cytokines, namely IL-17A-F [36]. A detailed description of the downstream molecular mechanisms activated upon IL-17 binding to its receptor is well beyond the scope of this review. Briefly, there are two signaling pathways, named canonical and non-canonical. The canonical pathway activates transcription factors such as MAPK, C/EBPβ, and NF-κB. Subsequent activation of these pathways results in increased in transcription of genes encoding chemokines, proinflammatory cytokines, and antimicrobial peptides. A hallmark of the non-canonical pathway is increased mRNA stability, especially mRNA encoding relatively unstable targets such as inflammatory mediators [38]. Altogether, both canonical and non-canonical pathways tend to promote the inflammatory response.

### IL-17 is ubiquitous in the tumor microenvironment and appears to play an important role in the pathobiology of solid tumors

IL-17 is an abundant cytokine in the tumor microenvironment [39]. Although ample evidence suggests that IL-17 is involved in tumor promotion and progression [40, 41], there have been conflicting reports on its role in hindering the growth of malignant cells [42], which has raised some controversy [43, 44]. Several mechanisms have been postulated, by which the IL-17 axis is involved in oncogenesis and cancer progression. First, IL-17 has been reported to be involved in the early stage of carcinogenesis. Indeed, it exerts its actions at the very beginning, contributing to the development of cancer. For instance, Gasmi et al. demonstrated that IL-17 promotes the transformation of premalignant cells, liver progenitor cells, into cancer stem cells in hepatocellular carcinoma in vitro. This observation was further confirmed in a mouse model [45]. Similarly, a role of IL-17 in tumor initiation has also been proposed in an animal pancreatic cancer model [46]. Pathological formation of blood vessels is one of the hallmarks of cancer, and IL-17 has been shown to promote angiogenesis by activating the STAT3/GIV pathway [47]. Moreover, it has the proclivity to promote self-renewal of cancer stem cells [48]. IL-17 also shapes the tumor microenvironment, making it more susceptible to cancer development. More precisely, one of the postulated mechanisms is the upregulation of PD-L1 on mesenchymal stromal cells within the tumor niche, which promotes tumor progression in a more immunosuppressive microenvironment [49]. To further support the role of IL-17 in tumor progression, it has been reported that chemoresistance in various cancers can be attributed, among others, to downstream mechanisms elicited by the IL-17 axis [50, 51]. Furthermore, as reported by Wang and colleagues, IL-17 increases the metastatic potential of tumors [52]. It can therefore be concluded that IL-17, taking into account tumor biology, is primarily associated with its growth, progression and resistance to therapy.

### IL-17 in MM—still more questions than answers

As mentioned above, majority of the evidence regarding the role of IL-17-related signaling in cancer comes from studies that examined its role in solid tumors. Hematological neoplasms have distinct characteristics and underlying pathogenesis. Therefore, the results of research on solid tumors cannot be directly translated to hemato-oncology. Nevertheless, although hematological malignancies are different, they share some similarities with solid tumors; hence, the results of the above-mentioned studies may provide a basis for further research in this field. MM is the second most common hematological malignancy. Despite recent progress, there is still room for improvement in both basic research and clinical outcomes. Although the vast majority of studies on the role of IL-17 in cancer have been conducted in solid tumors, there are also studies describing its role in MM. Given the important yet underappreciated role of IL-17 in the pathobiology of MM and the potential clinical implications, there is a need for a thorough review of the studies already conducted for the broader research community.

### IL-17 is a growth factor for MM cells, promotes myelomagenesis and facilitates disease progression

The proposed role of IL-17 in the initiation of various cancers has been mentioned above [45, 46]. Although the results from studies on solid tumors cannot be directly extrapolated to MM, they provide a basis for further studies on malignancies with different pathobiology.

Solid tumors, although different, share some similarities with hematological malignancies. One of them is the involvement of IL-17 in their initiation. According to Yang et al., IL-17 levels detected in BM are markedly elevated in NDMM patients compared to healthy controls without malignancy history (*p* < 0.001), strongly implying its role in myelomagenesis. Furthermore, IL-17 appears to promote extramedullary disease (EMD) by increasing angiogenesis. To clarify the exact mechanisms, it should be mentioned that masses of the skeletal EMD had the proclivity to increased microvessel density compared to BM of NDMM patients as well as to control group. Furthermore, the IL-17R was almost solely expressed in the endothelial cells of skeletal EMD, suggesting a key role of the IL-17 axis in its development [53]. Similar observations regarding the role of IL-17 in the promotion of MM and associated increased angiogenesis have been described elsewhere [54]; however, in this study, serum IL-17 levels were higher in NDMM compared to the control group, and this difference was not statistically significant [54]. Similarly, Lemancewicz et al. reported significantly elevated levels of IL-17A and IL-17E in MM patients in comparison with the healthy controls. Moreover, in accordance with the obtained results, plasma IL-17A concentration positively correlated with the percentage of plasma cells in trephine biopsy and serum lactate dehydrogenase level and was associated with a more advanced stage of the disease, suggesting its role in promoting MM growth. However, the percentage of plasma cells in trephine biopsy correlated negatively with plasma IL-17E levels, suggesting its not precisely defined role in the pathobiology of MM [55].

Calcinotto et al. conducted an interesting study in which they shed more light on the pathogenesis of MM initiation and its progression to fully developed disease in a mouse model. First, they demonstrated a causal relationship between changes in gut microbiota and the development of MM. Having established that *P. heparinolytica* accounts for the acceleration of the disease, the authors moved to identification of underlying mechanisms. The obtained results revealed that *P. heparinolytica* promotes the induction of IL-17-producing cells both within the BM and that IL-17 positively affects myelomagenesis, particularly in the early phase of MM in Vk*MYC mice. Based on the obtained results, it was hypothesized that IL-17 promotes smoldering MM (SMM) to MM transition. To support this hypothesis, the authors retrospectively analyzed BM samples from patients who quickly progressed to MM (within less than 3 years) and compared these data with those obtained from a cohort of SMM patients who did not progress to MM during the same time period. The analysis revealed that baseline IL-17 levels were significantly higher in patients who rapidly progressed. This supports the hypothesis that IL-17 is involved in the early stage of MM development, facilitating the progression of the disease from the premalignant stage. In addition, the results exposed the complicated interplay with eosinophils, suggesting their vital role in IL-17-mediated MM promotion [56]. Increased BM levels of IL-17 and percentage of Th17 cells compared to healthy controls provide further evidence for the involvement of the IL-17 axis in the development of MM [57].

BM in MM is highly immunosuppressive and permissive for MM cells what might be attributed to interaction between Th17 cells and BM T-regs. As mentioned above, BM in MM is characterized by elevated levels of IL-17 and increased percentage of Th17 cells. T-regs in MM to huge extent contribute to the development of permissive environment for MM [58] and are associated with inferior outcomes [59]. Moreover, having considered that Th17 cells and T-regs reciprocally stimulate each other [60], the interplay between these cells might, among others, result in MM promotion and progression.

### IL-17 axis promotes bone disease in MM via promoting osteoclastogenesis

In addition to directly stimulating MM growth, progression of SMM to MM, and EMD, IL-17 contributes to bone disease in MM. According to the study conducted by Noonan et al., plasma and BM levels of IL-17 were significantly elevated in MM patients compared to healthy controls. Furthermore, they demonstrated that the cytokine profile within BM niche shifts T cells into Th17 phenotype, and these cells predominate in the BM of MM patients. Moreover, IL-17 was a major contributor to lytic bone disease irrespective of percentage plasma cells in the biopsies and other clinical variables, including ethnicity, International Staging System, immunoglobulin subtype, or poor-risk chromosomal translocations [9]. Furthermore, IL-17 stimulates the osteoclast-like differentiation of immature dendritic cells. In this way, it mediates hyperactive osteoclastogenesis, resulting in excessive bone resorption activity. These effects were mediated, at least in an in vitro model, by IL17RA [61].

### The prognostic value of IL-17-related signaling

BM levels of IL-17 have also other clinical implications. Apart from its association with bone disease in MM, IL-17 has, at least to some extent, prognostic value. Furthermore, the percentage of Th17 cells within the BM was higher in stage III than in stage I + II MM, further indicating the clinical relevance of the IL-17 axis [62].

In addition, it was showed that higher ratio of IL-27:IL-17 in the BM was associated with a superior PFS (HR = 0.160; 95% CI:0.058–0.443; *p* < 0.001), implying that elevated levels of IL-17 negatively affect the prognosis of MM patients [63]. In another study, the number of Th17 cells within the BM at least partially correlated with disease status and therefore has prognostic value. The overall number of Th17 cells in patients with MM was shown to be significantly higher than in the healthy control group, although this range showed considerable fluctuation. More specifically, compared with the control group, the percentage of Th17 cells was significantly increased in NDMM patients who achieved partial remission and in the relapsed groups, but significantly decreased in patients who achieved complete remission. Moreover, NDMM patients with normal Th17 cell counts were significantly more likely to achieve complete remission [64]. Moreover, another study showed that the number of Th17 cells was significantly associated with serum lactate dehydrogenase levels, renal function and disease stage [65].

Bai et al. reported another observation regarding the prognostic value of IL-17-related signaling. An increase in the level of interferon regulatory factor 4 (IRF4) in the bone marrow along with an increased number of Th17 cells and elevated plasma IL-17 levels have been demonstrated in patients with MM compared to the control group (healthy volunteers). Further analysis revealed that the proportion of Th17 cells corresponded with the disease stage and this effect appeared to be mediated by IRF4 [66]. Analysis of the cytokine profile in 105 individuals with NDMM provided clinically relevant observations regarding the clinical course of MM and the cytokine profile. Both univariate and multivariate analyses showed that serum IL-17A levels > 4 pg/mlL along with serum IL-6 levels > 3 pg/mL and treatment regimens were independent prognostic factors for poor PFS and OS [67]. Another study confirming the association between IL-17 levels, disease stage, and response to the treatment was conducted by Li et al. More specifically, the results revealed that elevated IL-17 levels negatively influenced treatment response and disease stage [68].

## Therapeutic interference with IL-17-related signaling is a promising therapeutic approach

IL-17 signaling is crucial for the development and progression of MM. As mentioned above, IL-17 promotes the transition of SMM to MM and stimulates MM cell proliferation. Furthermore, it contributes to the bone disease and MM via promoting the generation of osteoclasts and has prognostic value. Therefore, therapeutic interference with the IL-17 axis seems to be a feasible and promising research and therapeutic approach, especially considering the fact that tumor suppressor molecules in MM, such as MiR-15a/16, inhibit myelomagenesis, among others, by reducing IL-17 expression [69].

Prabhala et al. demonstrated increased number of Th17 cells in the BM microenvironment in MM, which was accompanied by increased BM levels of Th17-related cytokines, including IL-17 and IL-23. The experimental study further demonstrated a promoting effect of IL-17 on MM, both in vitro and in a mouse model. Moreover, with potential clinical implications, the expression of IL-17 receptor (IL-17R) is higher on MM cells than normal plasma cells, and a monoclonal antibody directed against IL-17R inhibited MM cell proliferation in the presence of IL-17 [8]. Having established the latter, Prabhala and colleagues deepened and expanded the analysis in subsequent studies. It was clearly demonstrated that anti-IL-17A monoclonal antibody significantly reduced the proliferation rate of MM cells both in the presence and absence of bone marrow stromal cells. Furthermore, according to the obtained results, the presence of anti-IL-17A monoclonal antibody contributed to the downregulation of the osteoclasts. The above observations regarding the inhibition of MM growth via therapeutic interference with IL-17-related signaling were further demonstrated in a mouse model as described below [70]. Interestingly, everolimus inhibited the propensity of dendritic cells to acquire osteoclast activity by blocking IL-17A receptor-driven signals [71], which further supports the hypothesis of an important role of IL-17 in bone diseases in MM.

Results of our study revealed the upregulation of IL-17R in MM cells in patients who achieved deep and long-lasting response to Rd (LEN + dexamethasone) regimen compared to NDMM individuals. This strongly implies that MM cells, under the pressure exerted by LEN, aim to synthesize themselves to IL-17-related signal, presumably to evade the environmental pressure exerted by the drug. Furthermore, this provides another evidence that therapeutic interference with IL-17-related signaling may serve as an effective adjuvant immunotherapy therapy, especially in LEN-based treatment regimens, enhancing its cytotoxic effects on MM cells [10].

## Future perspectives and conclusions

Progress in the treatment of MM in recent years has significantly improved clinical outcomes for patients, with prolonged survival being a particular example. However, although much has been done, there is still room for improvement. Therefore, there is an urgent need to search for new molecules and new generations of compounds belonging to the currently used drug classes in the treatment of MM. In addition, basic research aimed at identifying potentially exploitable molecular pathways can provide a basis for the implementation of existing compounds used in various indications. One such potential new approach to treating MM is the IL-17 axis. Th17 cells and IL-17 itself have been shown to participate in myelomagenesis, accelerating the transition from MGUS to MM and promoting disease progression. Furthermore, there is emerging evidence that this signaling pathway contributes to EMD and, via osteoclast activation, to MM bone disease.

The alterations in IL-17-related signaling within malignant plasma cells, elicited the exposure anti-MM drugs, are still terra incognita. There is scarce evidence regarding how major drug classes affect the population of Th17 cells, IL-17 concentration and expression, and IL-17R receptor abundance on MM cells. To the best of our knowledge, the results of a study [10] conducted by our team report for the first time that expression of IL-17R increases as a result of long-lasting exposure to LEN in MM patients treated with Rd regimen who achieved deep response. The effect of PIs, monoclonal antibodies, bispecific antibodies, and CAR-T cells on the different components of this complex axis remain to be discovered and should therefore be investigated in future studies.

Monoclonal antibodies against IL-17 or IL-17R are currently approved for the treatment of autoimmune diseases such as psoriasis, psoriatic arthritis and ankylosing spondylitis [72, 73]. Despite preclinical rationale, these agents have never been tested in clinical trials for MM. Although there is emerging evidence that IL-17 targeted therapy might be clinically useful in MM, there are certain barriers which need to be tackled. In the first place, there is need for more preclinical research to explore the involvement of IL-17 axis in MM pathobiology as the preclinical rationale is essential for further advances in this field. Furthermore, the implementation of IL-17 antagonists in MM treatment requires clinical trials as none of this compounds is currently registered in this indication.

The introduction of IL-17 antagonists in MM further supports the fact that cytopenias and peripheral neuropathy are rarely reported as adverse events in this setting [74], in contrast to canonical drug classes used in MM such as protease inhibitors (PIs) and immunomodulatory drugs (IMIDs), which are often associated with myelosuppression, necessitating dosage modification or even treatment discontinuation [27, 28]. For instance, according to the results of phase III study of brodalumab for the treatment of ankylosis spondylitis cytopenias occurred in only 1.8% of participants depicting consistent safety profile over 68 week observation [75]. The implementation of these agents which might enhance anti-MM effects of commonly used treatment regimens yet without exacerbation of adverse events may improve response and prolong survival of MM patients.

Although there is emerging evidence that IL-17 targeted therapy might be clinically useful in MM, there are certain barriers which need to be tackled. In the first place, there is need for more preclinical research to explore the involvement of IL-17 axis in MM pathophysiology as the preclinical rationale is essential for further advances in this field. Furthermore, the implementation of IL-17 antagonists in MM treatment requires dedicated clinical trials as none of this compounds is registered in this indication. In addition, IL-17 antagonists are associated with an increased risk of infection, predominantly respiratory tract [75], which might also limit its application into MM treatment.

## Data Availability

Not applicable.
